# Prophylactic cranial irradiation in patients with small cell lung cancer in The Netherlands: A population-based study

**DOI:** 10.1016/j.ctro.2021.02.001

**Published:** 2021-02-12

**Authors:** Mathijs L. Tomassen, Mieke J. Aarts, Max Peters, Anne van Lindert, Dirk K.M. De Ruysscher, Joost J.C. Verhoeff, Peter S.N. van Rossum

**Affiliations:** a Department of Radiation Oncology, University Medical Center Utrecht, The Netherlands; b Netherlands Cancer Registry, Netherlands Comprehensive Cancer Organization, Utrecht, The Netherlands; c Department of Pulmonology, University Medical Center Utrecht, The Netherlands; d Department of Radiation Oncology (Maastro), GROW School for Oncology and Developmental Biology, Maastricht University Medical Centre+, Maastricht, The Netherlands

**Keywords:** Small cell lung cancer, Prophylactic cranial irradiation, Population-based, Survey, MRI

## Abstract

**Introduction:**

Controversy has arisen regarding the benefit of prophylactic cranial irradiation (PCI) in patients with small cell lung cancer (SCLC), particularly since the 2017 Takahashi trial publication that supports MRI surveillance in extensive-stage (ES-)SCLC. The primary aim of this study was to assess trends and determinants in PCI use over the years 2010–2018. A secondary aim was to determine contemporary practice considerations among radiation oncologists (ROs).

**Methods:**

A nationwide population-based cohort study was conducted using the Netherlands Cancer Registry data on all newly diagnosed SCLC patients (2010–2018). The change in PCI frequency over the years and determinants for PCI were analyzed using logistic regression models. Second, an online survey was performed among Dutch lung cancer ROs in 2020.

**Results:**

Among 10,264 eligible patients, 4,894 (47%) received PCI. Compared to 2010–2014, PCI use significantly decreased in 2017–2018 in ES-SCLC (OR 0.68, 95%CI 0.60–0.77) and LS-SCLC (OR 0.56, 95%CI 0.47–0.67). Incidence year, age, performance status, and thoracic radiotherapy were independent determinants for PCI. Among 41 survey participants, PCI was recommended always/sometimes/never by 22%/71%/7% in ES-SCLC and 54%/44%/2% in LS-SCLC. For ES-SCLC and LS-SCLC, 63% and 25% of ROs, respectively, confirmed influence of the Takahashi trial on PCI recommendations. Denial of such influence was associated with insufficient institutional MRI capacity.

**Conclusions:**

A significant declining trend of PCI use in both ES-SCLC and LS-SCLC was observed in The Netherlands since 2017. The Takahashi trial seems an explanation for this trend even in LS-SCLC, with differential influence of the trial depending on institutional MRI capacity. An alarming increase in practice variation regarding PCI was found which stresses the importance of ongoing trials.

## Highlights

• Prophylactic cranial irradiation (PCI) use declined in The Netherlands.
• Independent predictors for prescription of PCI were identified.
• An alarming increase of practice variation was observed.
• Alternative MRI surveillance is not strictly adhered to.

## 1 Introduction

With over 2,000,000 new cases per year, lung cancer is the most commonly diagnosed cancer worldwide (11.6% of the total cases), with the subgroup small cell lung cancer (SCLC) representing 13% of cases [1]. SCLC has a propensity of high metastatic spread reflected by a frequent occurrence of brain metastases (BM) at time of diagnosis of 16% and a 2-year cumulative incidence of 58% [2], [3]. It is more frequently staged as extensive stage disease (ES-SCLC) than limited stage disease (LS-SCLC) at the time of diagnosis (63% versus 37%) [4]. Accordingly, ES-SCLC has a poor prognosis with a 2-year overall survival (OS) of 13%, whereas LS-SCLC has a 2-year survival rate of 43% and 5-year survival rate of 25% [5], [6].

Prophylactic cranial irradiation (PCI) is conducted to reduce the incidence of BM in both ES-SCLC and LS-SCLC. An important fundament for PCI as treatment in LS-SCLC is the *meta*-analysis published by Aupérin et al. in 1999 [7]. The investigators showed a beneficial effect for the patients with LS-SCLC in complete remission treated with PCI versus no PCI on OS (relative risk [RR] = 0.85, 95% CI [0.73–0.99]) and on incidence of BM (RR = 0.48, 95% CI 0.38–0.60). In ES-SCLC, the first phase III randomized controlled trial (RCT) was the European EORTC trial published by Slotman et al. in 2007 [8]. Comparing PCI to no PCI in 143 versus 143 patients with ES-SCLC with a response after initial chemotherapy, the investigators concluded that PCI was beneficial in terms of the incidence of symptomatic BM (16.8% versus 41.3%, *p*=<0.001), disease free survival (median 14.7 versus 12.0 weeks, hazard ratio [HR] = 0.76, *p* = 0.02) and OS (median 6.7 versus 5.4 months, HR = 0.68, *p* = 0.003). After these two publications [7], [8], PCI has been part of the standard treatment recommendations for both ES-SCLC and LS-SCLC [9], [10].

Controversy has arisen regarding PCI in ES-SCLC since the Japanese trial by Takahashi et al. was published in 2017 [11]. This phase III RCT compared PCI followed by MRI surveillance to MRI surveillance alone in 113 versus 111 patients with ES-SCLC, who had shown any response to platinum-based doublet chemotherapy. Patients were only included if BM were excluded by brain MRI. This trial was terminated prematurely, as in interim analysis PCI did not result in a longer OS than the control group (median 11.6 versus 13.7 months, HR = 1.27, *p* = 0.094), and did not reveal a difference in progression free survival (median 2.3 versus 2.4 months, HR = 0.98, *p* = 0.75). However, the incidence of symptomatic BM (48% versus 69%, HR not reported, *p* < 0.0001) was beneficial regarding PCI.

Due to this controversy, the primary aim in the present study was to investigate the trends of PCI utilization in The Netherlands. Therefore, we assessed the trends in prescription of PCI over the years 2010–2018 in The Netherlands in a real-world population-based cohort and assessed which patient-, tumor-, and treatment-related characteristics determined the choice for PCI versus no PCI. A secondary aim was to determine the daily practice considerations of lung cancer radiation oncologists (ROs) in 2020 for prescribing PCI, in patients with ES-SCLC and LS-SCLC through a nationwide survey among ROs in the Netherlands.

## 2 Materials and Methods

Regarding the primary aim a nationwide population-based observational cohort study was approved by our institutional review board. The need for written informed consent was waived. For the secondary aim, an online survey was sent out to members of the National Platform for Radiotherapy for Lung Tumors with permission of their board, and data was handled anonymously.

### 2.1 Population-based cohort study

Details of patient-, tumor- and treatment-related data of all newly diagnosed cases of cancer in The Netherlands are continuously documented through a standardized procedure by trained data registration officers of the Netherlands Cancer Registry (managed by the Netherlands Comprehensive Cancer Organization) [12]. This data is recorded from the electronic medical records after notification from the national automated pathological archive (PALGA) [13]. All patients aged ≥18 with newly diagnosed SCLC of stage I-IV between January 2010 and December 2018 were included. Exclusion criteria were patients living abroad at time of diagnosis, brain metastases at time of diagnosis, no chemotherapy and tumors which were discovered at autopsy.

### 2.2 Variables

The main outcome parameter was administered PCI (yes/no). Analyzed variables at baseline were age, sex, incidence year (2010–2018), WHO performance status (0–4), tumor localization and lateralization, clinical TNM-stage, extent of disease (extensive or limited) and hospital type of diagnosis (academic hospital/top clinical hospital/general hospital). Studied treatment-related characteristics included hospital type of treatment (academic hospital/top clinical hospital/ general hospital), chemotherapy (yes/ no), thoracic radiotherapy (yes/no), and timing of chemotherapy versus thoracic radiotherapy (concurrent/sequential). Incidence year was categorized into time frames 2010–2014, 2015–2016 or 2017–2018 based on the first Japanese trial presentation at ASCO in 2014 [14] with subsequent related discussions in articles in 2015 and 2016 [15], [16], [17], [18], [19], and its final publication in May 2017 [11]. Due to modifications of the TNM classification system in 2017 (from 7th to 8th edition) clinical T-stage ‘cT3′, ‘cT4′ as well as clinical M−stage ‘cM1b’ and ‘cM1c' were merged for the purpose of this study. As such, all studies cTNM-stage categories complied with the 8th edition of the TNM classification system. Also, extent of disease (limited or extensive) was categorized, in accordance with the TNM 8th edition.

### 2.3 Statistical analysis

Comparing patients with and without administered PCI, continuous parameters were investigated using the independent-sample T-test and depicted as mean with standard deviation (SD). Ordered and non-ordered categorical parameters were compared using Mann-Whitney U and Chi-square tests. Trends in the use of PCI were plotted for ES-SCLC and LS-SCLC. Univariable logistic regression analysis was performed to study whether the change in frequency of PCI prescription significantly changed from time frame 2010–2014 to 2015–2016 and 2017–2018.

To adjust for potential confounders in the trend of PCI use, multivariable logistic regression models for the prediction of PCI use were built (separately for the ES-SCLC and LS-SCLC groups). First, missing data was considered to be missing at random and handled by multiple imputation using chained equations, creating 100 new datasets [20]. All modelling steps were pooled over these datasets. Second, potential effect modification of incidence year on the influence of other parameters for choosing PCI was studied using interaction terms in logistic regression models. All studied variables and a significant interaction term were entered into a full model, after which stepwise backward elimination based on Akaike’s Information Criterion (AIC) was used to exclude redundant variables [21]. The final models were presented as odds ratios (ORs) with 95% confidence intervals (CIs). Analyzes were performed using SPSS version 25.0 (IBM Corp, IBM SPSS Statistics for Windows, Armonk, NY) and R version 4.0.0 (‘mice’ and ‘rms’ packages). A p-value < 0.05 was considered statistically significant.

### 2.4 Nationwide survey among ROs

A pseudonymized online survey was sent out to all radiation oncologist members of the National Platform for Radiotherapy for Lung Tumors in June 2020. Respondents answered questions about demographics, influence of the Japanese RCT and decisions regarding PCI treatment for ES-SCLC and LS-SCLC [11]. Depending on given responses, a maximum of 37 questions could be answered (Supplemental Table 1). The Likert scale (1–5, low–high importance) was used to indicate the importance of a factor to advise PCI. If ≥ 75% of all the ROs rated it 4 (important) or 5 (very important) a factor was considered as important [22]. Fisher’s exact tests were conducted to investigate potential associations of demographics or MRI capacity with personal PCI recommendations or personal influence of the Japanese trial in PCI considerations for ES-SCLC and LS-SCLC [11]. Since some institutes had multiple respondents, sensitivity analysis was conducted for observed significant associations by equally weighting each institute.

## 3 Results

### 3.1 Population-based cohort study

A total of 15,564 patients with SCLC met the inclusion criteria. Patients with BM at baseline (n = 1,746) and patients without BM at baseline who received no chemotherapy (n = 3,554) were excluded. Among the 10,264 remaining patients eligible for analysis, 4,894 (47.4%) received PCI. Baseline patient-, tumor-, and treatment-related characteristics are presented in Table 1. Mean age in the PCI group was 64.7 years (±8.5) compared to 67.2 (±9.0) in the no-PCI group (*p* < 0.001). Other small (but statistically significant) differences between both groups were observed regarding sex, WHO performance status, and tumor localization. Larger differences between PCI and no-PCI groups were found for clinical M−stage (M1: 50% versus 71% respectively), extent of disease (ES-SCLC: 54% versus 74%, respectively), and thoracic radiotherapy (57% versus 19%, respectively).

**Table 1:** Baseline and treatment-related patient characteristics.

	Prophylactic cranial irradiation (n = 4,894)	No prophylactic cranial irradiation (n = 5,370)	*p* value
Age, mean (±SD)	64.7 ± 8.5	67.2 ± 9.0	<0.001*
Male sex	2,391 (49%)	2,847 (53%)	<0.001*
Incidence year			<0.001*
2010–2014	2,979 (61%)	2,841 (53%)	
2015–2016	1,069 (22%)	1,204 (22%)	
2017–2018	846 (17%)	1,325 (25%)	
WHO performance status			<0.001*
0–1	1,158 (24%)	1,219 (23%)	
2	160 (3%)	299 (6%)	
3–4	26 (<1%)	132 (2%)	
*Missing*	3,550 (73%)	3,720 (69%)	
Localization			0.029*
Main bronchus	879 (18%)	854 (16%)	
Lung upper lobe	2,186 (45%)	2,408 (45%)	
Lung middle lobe	218 (4%)	194 (4%)	
Lung lower lobe	1,035 (21%)	1,168 (22%)	
Lung overlapping	203 (4%)	236 (4%)	
*Missing*	373 (8%)	510 (9%)	
Lateralization			0.851
Left	2,036 (42%)	2,178 (40%)	
Right	2,688 (55%)	2,941 (55%)	
Both sides	5 (<1%)	5 (<1%)	
*Missing*	165 (3%)	246 (5%)	
Clinical T-stage			0.050
cT1	627 (13%)	630 (12%)	
cT2	759 (16%)	760 (14%)	
cT3-4	3,094 (63%)	3,397 (63%)	
*Missing*	414 (8%)	583 (11%)	
Clinical N-stage			0.066
cN0	326 (6%)	367 (7%)	
cN1	286 (6%)	266 (5%)	
cN2	2,053 (42%)	2,165 (40%)	
cN3	2,146 (44%)	2,433 (45%)	
*Missing*	83 (2%)	139 (3%)	
Clinical M−stage			<0.001*
cM0	2,449 (50%)	1,558 (29%)	
cM1a	308 (6%)	398 (7%)	
cM1b-c	2,137 (44%)	3,414 (64%)	
Stage of disease			<0.001*
Limited	2,276 (46%)	1,404 (26%)	
Extensive	2,608 (54%)	3,959 (74%)	
*Missing*	10 (<1%)	7 (<1%)	
Hospital of diagnosis			0.435
General hospital	1,980 (40%)	2,132 (40%)	
Top clinical/academic hospital	2,914 (60%)	3,238 (60%)	
Hospital of treatment			0.639
General hospital	1,915 (39%)	2,077 (39%)	
Top clinical/academic hospital	2,979 (61%)	3,293 (61%)	
Thoracic treatment			<0.001*
No radiotherapy	2,085 (43%)	4,377 (81%)	
Sequential radiotherapy	1,228 (25%)	411 (8%)	
Concurrent radiotherapy	1,581 (32%)	582 (11%)	

**Fig. 1:**
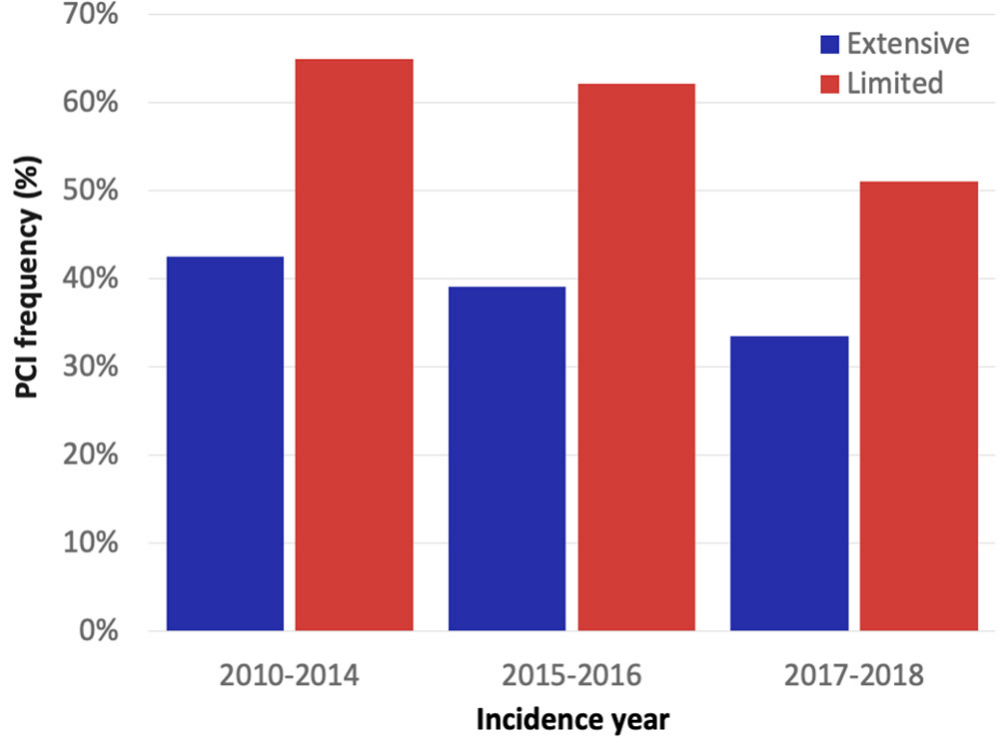
Frequency of prophylactic cranial irradiation (PCI) use among all patients with small cell lung cancer without brain metastasis who underwent chemotherapy in The Netherlands over time.

Over time, the use of PCI decreased from 43 to 35% in ES-SCLC and from 64 to 50% in LS-SCLC (Fig. 1). Univariable analysis in the 6,567 ES-SCLC patients showed that compared to 2010–2014 the odds of receiving PCI significantly decreased in time frame 2015–2016 (unadjusted OR 0.87, 95% CI: 0.77–0.98, *p* = 0.026), and even further in 2017–2018 (unadjusted OR 0.68, 95% CI: 0.60–0.77, *p* < 0.001). In the 3,680 LS-SCLC patients, compared to 2010–2014 the odds of receiving PCI was not significantly different in 2015–2016 (unadjusted OR 0.88, 95% CI: 0.75–1.05, *p* = 0.149), but was significantly decreased in 2017–2018 (unadjusted OR 0.56, 95% CI: 0.47–0.67, *p* < 0.001).

In multivariable analysis, entering all studied determinants plus 1 significant interaction term (clinical N-stage in interaction with incidence year) and subsequently eliminating redundant determinants resulted in two final models for predicting PCI use (in ES-SCLC and LS-SCLC separately; Table 2). Adjusted for potential confounders and effect modifiers, incidence year remained significantly related to the chance of receiving PCI. In both ES-SCLC and LS-SCLC patients, compared to 2010–2014 the odds of receiving PCI was significantly reduced in 2015–2016 (adjusted OR 0.70 and 0.75, respectively) and even further in 2017–2018 (adjusted OR 0.52 and 0.47, respectively). Besides incidence year, in both ES-SCLC and LS-SCLC patients, main drivers for receiving PCI included younger age, better WHO performance status and thoracic radiotherapy. In ES-SCLC, clinical stages N2-3 and M1 were additional independent predictors of PCI prescription.

**Table 2:** Multivariable logistic regression models predicting PCI use in ES-SCLC and LS-SCLC.

Variable	Extensive stage			Limited stage		
	n	OR (95% CI)	*p* value	n	OR (95% CI)	*p* value
Age	6,567	0.98 (0.97–0.99)	<0.001*	3,680	0.96 (0.95–0.97)	<0.001*

Performance score						
WHO 0–1	5,076†	Ref		3,089†	Ref	
WHO 2	1,042†	0.62 (0.50–0.76)	<0.001*	459†	0.77 (0.57–1.04)	0.093
WHO 3–4	449†	0.29 (0.21–0.42)	<0.001*	132†	0.44 (0.24–0.80)	0.008*

Incidence year						
2010–2014	3,574	Ref		2,240	Ref	
2015–2016	1,494	0.70 (0.61–0.80)	<0.001*	768	0.75 (0.62–0.91)	0.004*
2017–2018	1,499	0.52 (0.46–0.60)	<0.001*	672	0.47 (0.38–0.57)	<0.001*

Thoracic radiotherapy						
None	5,589	Ref		867	Ref	<0.001*
Sequential	761	7.06 (5.84–8.54)	<0.001*	877	10.3 (8.22–13.0)	
Concurrent	217	4.44 (3.19–6.18)	<0.001*	1,936	8.41 (6.88–10.3)	<0.001*
Type of hospital of treatment						
General	2,626	Ref		1,362	Ref	
Top clinical or academic	3,941	0.93 (0.83–1.03)	0.164	2,318	0.91 (0.78–1.07)	0.264

Clinical T-stage						
cT1	552	Ref		826	Ref	
cT2	942	1.09 (0.85–1.39)	0.483	735	1.07 (0.84–1.37)	0.567
cT3-4	5,073	1.11 (0.91–1.37)	0.308	2,119	1.00 (0.82–1.22)	0.992

Clinical N-stage						
cN0	248†	Ref				
cN1	215†	1.15 (0.75–1.75)	0.521	-	-	
cN2	2,422†	1.38 (1.02–1.87)	0.036*	-	-	
cN3	3,682†	1.74 (1.29–2.35)	<0.001*	-	-	

Clinical M−stage						
cM0	410	Ref		-	-	
cM1a	644	1.61 (1.19–2.18)	0.002*	-	-	
cM1b-c	5,513	1.43 (1.10–1.85)	0.008*	-	-	

CI: Confidence interval. OR: Odds ratio. PCI: Prophylactic cranial irradiation. *: Statistically significant different odds for PCI compared to the reference group (p < 0.05). †: Numbers include imputed data according to 1 (of 20) imputation set(s).

### 3.2 Nationwide survey among ROs

A total of 65 ROs were invited to participate in the online survey, of which 41 (63%) respondents completed the survey. Eighteen (86%) of 21 invited institutes participated. The results regarding daily practice of ROs for PCI in SCLC are presented in Table 4. Detailed results are provided in Supplemental Tables 1 and 2. In ES-SCLC, in daily practice, 9 (22%) of the ROs always recommend PCI, whereas 29 (71%) sometimes and 3 (7%) never recommend PCI. In LS-SCLC, 22 (54%) always recommend PCI, whereas 18 (44%) sometimes and 1 (2%) never recommends PCI. In 96% of respondents treatment teams, MRI during follow-up was only made if neurological symptoms were prevalent which suggested brain metastases in patients with SCLC.

For ES-SCLC and LS-SCLC, 63% and 25% of ROs confirmed that their daily practice regarding PCI was influenced by the results of the Japanese trial [11]. A statistically significant association was observed between having insufficient logistical capacity for routine and repeated brain-MRI, and reporting a lack of an influence of the Japanese trial results on PCI prescription in LS-SCLC patients (Supplemental Table 2.4) [11]. These findings were concordant after equalizing weights per institute in sensitivity analysis (Supplemental Table 2.5).

## 4 Discussion

Controversy has arisen since the Japanese trial by Takahashi et al. regarding PCI in SCLC was presented [11]. Therefore, we conducted a nationwide population-based observational cohort study of all SCLC patients diagnosed between 2010 and 2018 in The Netherlands. We demonstrated a significant declining trend in PCI use for patients with SCLC since 2015. The association of incidence year with PCI use was independent of other important determinants including age, WHO performance score, disease stage, and thoracic radiotherapy. This suggests an external cause in those years not related to patient or tumor characteristics. We suggest that the results of the Japanese RCT presented at ASCO in 2014 [14], commented on in 2015 and 2016 [15], [16], [17], [18], [19] and published in 2017 played a major role in the observed paradigm shift [11]. This was supported by the additional national survey study reported here, in which 64% of lung cancer ROs in The Netherlands reported awareness of the RCT results from 2015 to 2017 and 63% stated that this RCT influenced their policy regarding PCI in ES-SCLC, whereas 25% stated this also for LS-SCLC.

Identified factors independently determining PCI prescription in both ES-SCLC and LS-SCLC in the 2010–2018 cohorts were younger age, better WHO performance status and use of thoracic radiotherapy. These results are in line with a recently reported prediction model in ES-SCLC patients from the United States that reported age (≥65 versus < 65 years, OR = 0.65, *p* = 0.003) and Charlson Comorbidity Score (≥1 vs. 0, OR 0.76, *p* = 0.006) as determinants for PCI [23]. In further support, the European Society for Medical Oncology (ESMO) clinical practice guidelines and Dutch guidelines recommend to take PCI in consideration in patients with a good WHO performance status and after any response to chemotherapy [9], [10]. As such, these are important factors to adjust for in analyzing new trends in PCI use. To our knowledge, before this current study no previous study assessed real-world determinants for PCI use in LS-SCLC.

Results of our 2020 survey among ROs in The Netherlands on current practice regarding PCI in SCLC confirmed the growing reluctance in performing routine PCI. Among respondents, noted reasons for refraining from PCI in order of importance based on Likert scale included patient preference, WHO performance status ≥ 2, availability of brain-MRI, late term neurotoxicity of PCI, pre-existent cognitive diseases and no response to induction chemotherapy. Also, 63% of respondents confirmed that the Japanese RCT influenced their decision-making for PCI in ES-SCLC [11]. This is in line with the results of a recent survey among radiation oncologists (n = 431, response rate 12.6%) conducted in the United States [24], in which a decline of 28% (95% CI: 25%–31%, *p* < 0.001) was seen for routinely offering PCI to ES-SCLC patients after the Japanese RCT [11]. In the US survey, also a trend of increased application of MRI surveillance in patients with ES-SCLC was noted.

The Japanese trial concluded to apply MRI surveillance instead of PCI [11]. Remarkably, our nationwide survey shows that 63% of the ROs indicate their treatment team does not perform a baseline MRI of the brain. In addition, 96% of the ROs and their teams do not routinely perform brain-MRI during follow-up in any stage of SCLC. Taken together, despite the fact that the ROs indicate influence of the Japanese trial for ES-SCLC (63%) and LS-SCLC (25%) on recommending PCI, this has not been accompanied by a shift towards routine use of brain-MRI. The observed association between (lack of) MRI capacity and its (lack of) routine application in SCLC suggests that factors other than patient- or tumor-related factors could play a role in inter-institutional differences in PCI decision-making.

Interestingly, although the Japanese trial was performed in ES-SCLC patients [11], our population-based study suggests extrapolation of the trial findings in LS-SCLC patients, with an adjusted reduction in the odds for PCI of 25% and 43%, respectively in time frames 2015–2016 and 2017–2018 compared to 2010–2014. In addition, 25% of the ROs in our survey mentioned that the results of the Japanese trial influenced their decision-making in prescribing PCI for LS-SCLC patients [11]. This raises the question whether the propensity to more often refrain from PCI is justified at all in LS-SCLC. The main driver for PCI in LS-SCLC was the *meta*-analysis of Aupérin et al. published in 1999 of 7 trials that enrolled patients from 1977 to 1995 [7]. This study recommended PCI as standard of care for LS-SCLC patients with a complete response on a chest X-ray after induction chemotherapy. An important concern about the generalizability of these historical trials (next to outdated radiation treatment techniques) includes the lack of imaging options (MRI or even CT) to diagnose brain metastases at that time, no contemporary staging, different treatment strategies and response evaluation based on a plain X-ray of the thorax. No prospective studies on PCI in LS-SCLC have been performed since then and subsequent 15 retrospective studies (including 10,900 patients) demonstrate conflicting results on both BM and OS outcomes (Table 3). Among these inconclusive studies, most studies did not perform routine brain-MRI at baseline to exclude BM. In fact, in the LS-SCLC studies that did report baseline MRI in (all or some) patients, 4 out of 6 studies found no significant improvement in BM incidence after PCI [18], [25], [26], [27] and 3 out of 4 studies observed no OS benefit [27], [28], [29].

**Table 3:** Overview of studies regarding PCI in LS-SCLC.

	Design	Baseline MRI	Response to chemo	n	PCI%	MRI SA	BM Incidence		
							PCI	No PCI	Significance (95% CI)
*Resected LS-SCLC*									
Bischof 2007 [32]	Retrospective	NR	NR	39	54%	NR	0%^c^	22%^c^	NR
Zhu 2014 [33]	Retrospective	Yes	NR	193	35%	No	9,0%^c^	22%^c^	*p =* 0.02
Yokouchi 2015 [34]	Retrospective	NR	NR	156	8.3%	NR	7,7%^c^	NR	NR
Xu 2017 [35]	Retrospective	NR	NR	349	33%	NR	13%^c^	23%^c^	*p* < 0.01

*Non-resected LS-SCLC*									
Patel 2009 [36]	Retrospective	No	NR	7,995	8.4%	NR	NR	NR	NR
Nakahara 2015 [37]	Retrospective	NR	PR/CR	40	45%	NR	33%^c^	50%^c^	*p* = 0.29
Ozawa 2015 [25]	Retrospective	Yes*	PR/CR	124	23%	NR	46%^a^	31%^a^	*p* = 0.30
Choi 2017 [29]	Retrospective	Yes*	Any	280	32%	NR	25%^c^	39%^c^	*p* = 0.01
Mamesaya 2018 [26]	Retrospective	Yes	PR/CR	38	50%	NR	24%^c^	27%^c^	*p* = 0.40
Farris 2019 [38]	Retrospective	NR	≥SD	92	42%	NR	32%^c^	29%^c^	*p* = 0.66
Pezzi 2020 [27]	Retrospective	Yes	Any	168	84	NR	11%	20%	HR = 0.51 (0.24–1.10)

*Both LS-SCLC and ES-SCLC*									
Auperin 1999 [7]	Meta-analysis	NR	CR	847	55%	No	33%^b†^	59%^b†^	RR = 0.46 (0.38–0.57)
Schild 2012 [39]	Pooled analysis	No	≥SD	421	80%	No	NR	NR	NR
Rule 2015 [28]	Pooled analysis	Yes*	≥SD	84	76%	No	NR	NR	NR
Nicholls 2016 [18]	Retrospective	Yes*	Any	74	43%	No	9%	19%	*p* = 0.33

^a^: 2-year cumulative incidence. ^b^: 3-year cumulative incidence. ^c^: Total cumulative incidence. BM: Brain metastases CI: Confidence interval. CR: Complete response. HR: Hazard ratio. MRI SA: MRI surveillance approach. NR: Not reported. PCI: Prophylactic Cranial Irradiation. PR: Partial response. RR: Relative risk. SD: stable disease. ^†^: total patients *: not all patients received MRI.

**Table 4:** Summary of national survey results in The Netherlands.

Participants
Number: 41 (63% of invited)
Hospital: academic (56%) or non-academic (44%)
Awareness of Takahashi trial: before 2015 (5%), 2015–2017 (64%), 2018-present (29%) or not aware (2%)
**Extensive stage small cell lung cancer (ES-SCLC)**
Recommend PCI: always (22%), sometimes (71%) or never (7%)
Influence of Takahashi on daily practice: yes (63%) or no (37%)
Sufficient MRI capacity among ‘yes’ (75%) vs. among ‘no’ (25%); *p* = 0.094
Important factors for omitting PCI*:
WHO performance status ≥ 2
Pre-existent cognitive disorders
Patient wish
No response to induction chemotherapy
Standard pre-chemotherapy MRI: yes (37%) or no (63%)
Standard post-chemotherapy MRI: yes (7%) or no (93%)
**Limited stage small cell lung cancer (LS-SCLC)**
Recommend PCI: always (54%), sometimes (44%) or never (2%)
Influence of Takahashi on daily practice: yes (75%) or no (25%)
Sufficient MRI capacity among ‘yes’ (66%) vs. among ‘no’ (34%); *p* = 0.007
Important factors for omitting PCI*:
WHO performance status ≥ 2
Pre-existent cognitive disorders
Patient wish
Availability of brain MRI
Neurotoxicity of PCI
**Other question**
Performs a brain-MRI during follow up in SCLC: no (96%), yes, no matter use of PCI (2%), yes, only with use of PCI (2%)

*: Based on Likert scale 1–5 (≥75% of participants scored ‘important’ or ‘very important’).

The survey finding that of Dutch ROs 71% sometimes and 7% never recommend PCI in patients with ES-SCLC, while 44% sometimes and 2% of the ROs never recommend PCI regarding patients with LS-SCLC, suggests that no uniform treatment policy regarding PCI for neither ES-SCLC nor LS-SCLC exists within The Netherlands. Therefore, a modern-day trial (including at least a baseline MRI of the brain and an MRI surveillance approach) would be desirable, which is acknowledged by 85% of the survey respondents in the current study. To this regard, results of the recently embarked SWOG randomized phase III MAVERICK trial in the United States investigating MRI surveillance alone versus MRI surveillance and PCI are eagerly awaited [30]. In Europe, the EORTC established a trial to assess PCI versus MRI surveillance in SCLC patients (PRIMALung trial) [31].

A few limitations of our study should be considered. First, as the aim was to evaluate the trends in PCI use and its determinants in a nationwide population-based cohort, no survival outcomes were studied nor available. However, studying survival outcomes would have likely resulted in biased results through confounding-by-indication. Second, although the trend in PCI use over time was adjusted for known confounders, some unavailable or unknown confounders may have been missed. Third, the number of ROs in the survey (n = 41) was too limited to discern subtle differences or perform subgroup analyses. However, with a response rate of 63% of all lung cancer ROs in The Netherlands, we believe the survey is a fair representation of current practice. The study is strengthened by providing real-world modern-day clinical data of all patients with SCLC as well as contemporary expert opinions.

In conclusion, a declining trend of PCI prescription in patients with ES-SCLC and LS-SCLC is observed. Based on our multivariable logistic regression model and a nationwide survey, it seems likely that the Japanese trial explains this declining trend [11]. Remarkably, the Japanese trial (which included ES-SCLC patients) seems to be extrapolated to patients with LS-SCLC in current practice. Hitherto, no consensus about PCI use in patients with SCLC is reached. Therefore, further studies are necessary to reach a clear treatment policy.

## Funding

No external funding source was involved in this investigation.

## Declaration of Competing Interest

The authors declare that they have no known competing financial interests or personal relationships that could have appeared to influence the work reported in this paper.
