# Supplementary material for: Prophylactic cranial irradiation in patients with small cell lung cancer in The Netherlands: A population-based study
Source: Clin Transl Radiat Oncol. 2021 Feb 12;27:157–63. doi: 10.1016/j.ctro.2021.02.001 (PMC7903055; doi:10.1016/j.ctro.2021.02.001)
Supplement: Supplementary data 1 [file mmc1.docx]

| **Supplementary Table 1** Complete survey results (questions translated from Dutch) |  |  |
| --- | --- | --- |
| **Demographic characteristic** | **No. (%)** | **Mean** |
| In what type of hospital do you practice?  Academic hospital  Non-academic hospital  Other | 23 (56)  18 (44)  0 (0) |  |
| Including yourself, how many radiation oncologists are working at your location?  2-10  11-20  >20 | 10 (25)  12 (29)  19 (46) |  |
| Including yourself, how many lung cancer radiation oncologists are working at your location?  1-2  3-4  5-6  >6 | 5 (12)  7 (17)  18 (44)  11 (27) |  |
| Since the last 2 years, did you conduct any research in small cell lung cancer?  Yes  No | 8 (20)  33 (80) |  |
| How many years of clinical experience do you have as a radiation oncologist?  0-5  6-10  11-20  21-30  >30 | 5 (12)  12 (29)  13 (32)  8 (20)  3 (7) |  |
| With treatment of how many lung cancer patients are you involved yearly?  1-10  11-20  >20 | 1 (2)  1 (2)  39 (96) |  |
| In how many hospitals do you participate in the multidisciplinary lung tumor board meeting?  1  2  >2 | 10 (24)  11 (27)  20 (49) |  |
| Which part of your daily clinical practice is related to patients with lung cancer?  1-10%  11-30%  31-50%  >50% | 1 (2)  9 (22)  18 (44)  13 (32) |  |
| How many patients with SCLC do you treat annually?  1-10  >10 | 21 (51)  20 (49) |  |
| In which year did you take note of the Japanese phase 3 randomized controlled trial by Takahashi (2017) which demonstrated that PCI has no beneficial value compared to MRI surveillance in extensive stage small cell lung cancer?  I did not take note of this study  Before the end of 2014  2015-2017  2018-present | 1 (2)  2 (5)  26 (64)  12 (29) |  |

| **Extensive stage small cell lung cancer (ES-SCLC)** | **No. (%)** | **Mean** |
| --- | --- | --- |
| Did the results of the Takahashi (2017) trial influence your daily patterns regarding PCI for patients with ES-SCLC? (n=40)  Yes  No  Explanation of yes or no (simplified)  Yes  “More use of MRI during follow-up”  “Shared decision making”  “Following Dutch guidelines”  “Less PCI for elderly patients”  “More evidence for MRI surveillance”  “Very reluctant with PCI”  “More MRI, especially during the covid-19 pandemic”  No  “Most important is to prevent brain metastases”  “Change of setting with baseline brain MRI”  “Following Dutch guidelines”  “Current policy based on EORTC trial ^8^”  “Lack of evidence for MRI surveillance”  “Lack of MRI capacity”  “Regional pulmonologists are not prone to MRI surveillance”  “Shared decision making has always been our policy” | 25 (63)  15 (37)  12 (48)  5 (20)  2 (8)  2 (8)  2 (8)  1 (4)  1 (4)  4 (27)  1 (7)  2 (13)  1 (7)  2 (13)  2 (13)  2 (13)  1 (7) |  |
| Do you recommend PCI in daily practice to (a part of) patients with ES-SCLC?  Yes, always  Yes, sometimes  No, never | 9 (22)  29 (71)  3 (7) |  |
| Please indicate for the next 9 options how important you consider the factor to not advise PCI in a patient with ES-SCLC (Likert scale 1-5, low-high importance) (n=32)  Other  “Patient’s wish”  “Very extensive burden of disease”  WHO performance status ≥ 2  Pre-existent cognitive disorders  No response to induction chemotherapy  Evidence to perform MRI surveillance as an alternative to PCI  Multiple comorbidities  Medical history with cardiovascular accident or transient ischemic attack  Neurotoxicity of PCI  Age > 70 years  Availability of brain-MRI at time of staging | 8 (25)  5 (67)  3 (33) | 4.9  4.8  4.2  4.1  3.5  3.4  3.3  3.3  3.1  3.1 |
| In ES-SCLC patients with what type of radiological response (RECIST 1.1) to induction chemotherapy do you recommend PCI? (multiple options possible)  Stable disease  Minor response  Partial response  Complete response  I do not offer PCI, regardless response  Do you take the thoracic tumor response of a patient with ES-SCLC into account in the decision for PCI? (n=38)  Yes  No | 7 (17)  18 (44)  33 (81)  31 (76)  3 (7)  32 (78)  6 (22) |  |

|  |  |  |
| --- | --- | --- |
| As a radiation oncologist, did you experience a reduction in the number of referrals by the pulmonologists for PCI in ES-SCLC patients in the past 3-5 years?  Yes  No | 18 (44)  23 (56) |  |
| Is there sufficient capacity in your (referring) hospital to conduct a brain-MRI after induction chemotherapy and in the follow-up of ES-SCLC patients?  Yes  No | 25 (61)  16 (39) |  |
| Before starting chemotherapy, does your treatment team perform a baseline brain-MRI in patients with ES-SCLC who are neurologically asymptomatic?  Yes  No | 15 (37)  26 (63) |  |
| After treatment with chemotherapy (whether or not combined with thoracic radiotherapy), does your treatment team perform a brain-MRI in patients with ES-SCLC who are neurologically asymptomatic?  Yes  No | 3 (7)  38 (93) |  |

| **Limited stage small cell lung cancer (LS-SCLC)** | **No. (%)** | **Mean** |
| --- | --- | --- |
| Did the results of the Takahashi (2017) trial influence your daily patterns regarding PCI for patients with LS-SCLC? (n=40)  Yes  No  Explanation of yes and no (simplified)  Yes  “More MRI instead of PCI”  “Patient wish”  “Conceptually the same situation as ES-SCLC”  “Very reluctant with PCI”  “With LS-SCLC, there is hope for a smaller risk of brain metastases and therefore   the chance of unnecessary treatment is greater”  “Yes, but less compared with ES-SCLC”  No  “Takahashi concerned ES-SCLC patients”  “PCI in LS-SCLC has been the standard for years”  “Lack of MRI capacity”  “Most important is to prevent brain metastases”  “Better prognosis”  “Better survival”  “Criticism for Takahashi trial”  “Enough trials to demonstrate the added value of PCI”  “Following Dutch guidelines”  “MRI surveillance is for many patients no alternative because of claustrophobia and   anxiety for more MRI’s”  “Regional pulmonologists are not prone to MRI surveillance” | 10 (25)  30 (75)  4 (40)  2 (20)  1 (10)  1 (10)  1 (10)  1 (10)  14 (48)  4 (14)  3 (10)  2 (7)  1 (3)  1 (3)  1 (3)  1 (3)  1 (3)  1 (3)  1 (3) |  |
| Do you recommend PCI in daily practice to (a part of) your patients with LS-SCLC?  Yes, always  Yes, sometimes  No, never    Please indicate for the next 10 options how important you consider the factor to not advise PCI to a patient with LS-SCLC (Likert scale 1-5, low-high importance) (n=19)  Other  “Patient wish”  WHO performance status ≥ 2  Pre-existent cognitive disorders  Availability of brain-MRI at time of staging  Neurotoxicity of PCI  No response to induction chemotherapy  Evidence to perform MRI surveillance as an alternative to PCI  Medical history with cardiovascular accident or transient ischemic attack  Multiple comorbidity  Uncertainty about the percentage of survival gain  Age > 70 years | 22 (54)  18 (44)  1 (2)  5 (100) | 5.00  4.7  4.3  4.3  4.3  3.9  3.8  3.6  3.6  3.3  3.3 |
| As a radiation oncologist, did you experience a reduction in the number of referrals by the pulmonologists for PCI in LS-SCLC patients in the past 3-5 years?  Yes  No | 9 (22)  32 (78) |  |
| Is there sufficient capacity in your (referring) hospital to conduct a brain-MRI after induction chemotherapy and in the follow-up of ES-SCLC patients?  Yes  No | 27 (66)  14 (34) |  |
| Before starting chemotherapy, does your treatment team perform a baseline brain-MRI in patients with LS-SCLC who are neurologically asymptomatic?  Yes  No | 37 (90)  4 (10) |  |
| After treatment with chemotherapy (whether or not combined with thoracic radiotherapy), does your treatment team perform a brain-MRI in patients with LS-SCLC who are neurologically asymptomatic?  Yes  No | 4 (10)  37 (90) |  |

| **Other questions** | **No. (%)** | **Mean** |
| --- | --- | --- |
| Does your treatment team perform a standard brain-MRI during follow-up in patients with any stage of SCLC?  Yes, no matter the use of PCI  Yes, only with the use of PCI  No, only if neurological symptoms are prevalent which suggest brain metastases  Which frequency is advised by your treatment team? (n=2)  Every month (possibly decreasing)  Every three months (possibly decreasing)  Every six months (possibly decreasing)  Other | 1 (2)  1 (2)  39 (96)  0 (0)  2 (100)  0 (0)  0 (0) |  |
| Do you consider an additional randomized study for SCLC patients (any stage) regarding PCI versus MRI surveillance?  Yes  No  Which type of SCLC? (n=35)  Extensive – no; Limited – yes  Extensive – yes; Limited – no  Extensive – yes; Limited – yes | 35 (85)  6 (15)  7 (20)  1 (3)  27 (77) |  |
| Would you be willing to participate with your medical center in an additional randomized study for SCLC patients (any stage) regarding PCI versus MRI surveillance?  Yes  No  Which type of SCLC? (n=37)  Extensive – no; Limited – yes  Extensive – yes; Limited – no  Extensive – yes; Limited – yes | 37 (90)  4 (10)  8 (22)  2 (5)  27 (73) |  |
| Please indicate for the next 7 options how important you consider the factor to improve the prognosis (both survival and reduction of adverse events) of patients with SCLC (Likert scale 1-5, low-high importance).  Improvement of systemic therapy which also reduces the incidence of brain   metastases  Techniques to reduce the neurocognitive side effects of PCI  Improvement of supportive therapy  Improvement of systemic therapy that can be given in conjunction with thoracic   radiotherapy  Techniques to reduce side effects of thoracic radiotherapy  Improvement of systemic therapy that can be given in conjunction with PCI  Dose escalation or intensification of thoracic radiotherapy |  | 4.3  3.7  3.7  3.6  3.5  2.9  2.8 |
| Do you have any other suggestion to improve the prognosis of patients with SCLC?  Yes  No  “Alternatives for PCI“  “Alternatives instead of PCI in LS-SCLC patients, for example single fraction   stereo on multiple brain metastases”  “Patient with anti-hu antibodies more at risk for neurotoxicity”  “Role of SRS and MRI follow up in SCLC”  “Use of dexamethasone during PCI to reduce neurocognitive harm + Timing of PCI   + HA-PCI and Memantine” | 5 (12)  36 (88)  1 (20)  1 (20)  1 (20)  1 (20)  1 (20) |  |

| **Supplemental Table 2.1** Associations of demographic factors and MRI capacity with PCI recommendation in ES-SCLC | | | | |
| --- | --- | --- | --- | --- |
|  | **No. (%)** |  | |  |
|  |  | **Recommendation of PCI in  ES-SCLC (n=41)** | |  |
| **Factor** | **Respondents** | **Yes, always** | **No/not always** | ***p* value** |
| Type of hospital  Academic  Non academic | 23 (56)  18 (44) | 4 (17)  5 (28) | 19 (83)  13 (72) | 0.471 |
| No. of ROs at location  ≤20  >20 | 22 (54)  19 (46) | 5 (23)  4 (21) | 17 (77)  15 (79) | 1.000 |
| No. of lung ROs at location  ≤4  >4 | 12 (29)  29 (71) | 4 (33)  5 (17) | 8 (67)  24 (83) | 0.408 |
| Years of experience  ≤10 years  >10 years | 17 (42)  24 (58) | 3 (18)  6 (25) | 14 (82)  18 (75) | 0.711 |
| Involved in SCLC research last 2 years  Yes  No | 8 (20)  33 (80) | 0 (0)  9 (27) | 8 (100)  24 (73) | 0.164 |
| NSCLC/SCLC patients treated annually  ≤20  >20 | 2 (5)  39 (95) | 0 (0)  9 (23) | 2 (100)  30 (77) | 1.000 |
| Number of multidisciplinary boards participating  ≤2  >2 | 21 (51)  20 (49) | 5 (24)  4 (20) | 16 (76)  16 (80) | 1.000 |
| Percentage of daily practice associated with lung cancer  ≤50%  >50% | 28 (68)  13 (32) | 6 (21)  3 (23) | 22 (79)  10 (77) | 1.000 |
| Number of patients treated with SCLC annually  1-10  >10 | 21 (51)  20 (49) | 3 (14)  6 (30) | 18 (86)  14 (70) | 0.277 |
| Year of taking note of Takahashi trial  ≤2017  2018-present | 28 (68)  12 (29) | 7 (25)  1 (8) | 21 (75)  11 (92) | 0.396 |
| Sufficient capacity of brain MRI  Yes  No | 25 (61)  16 (39) | 6 (24)  3 (19) | 19 (76)  16 (81) | 1.000 |
| Pre-chemotherapy brain MRI  Yes  No | 15 (37)  26 (63) | 1 (7)  8 (31) | 14 (93)  18 (69) | 0.119 |
| Post-chemotherapy brain MRI  Yes  No | 3 (7)  38 (93) | 0 (0)  9 (24) | 3 (100)  29 (76) | 1.000 |

| **Supplemental Table 2.2** Associations of demographic factors and MRI capacity with PCI recommendation in LS-SCLC | | | | |
| --- | --- | --- | --- | --- |
|  | **No. (%)** |  | |  |
|  |  | **Recommendation of PCI in LS-SCLC (n=41)** | |  |
| **Factor** | **Respondents** | **Yes always** | **No/not always** | ***p* value** |
| Type of hospital  Academic  Non academic | 23 (56)  18 (44) | 12 (52)  10 (56) | 11 (48)  8 (44) | 0.540 |
| No. of ROs at location  ≤20  >20 | 22 (54)  19 (46) | 12 (55)  10 (53) | 10 (45)  9 (47) | 0.576 |
| No. of lung ROs at location  ≤4  >4 | 12 (29)  29 (71) | 7 (58)  15 (52) | 5 (42)  14 (48) | 0.485 |
| Years of experience  ≤10 years  >10 years | 17 (42)  24 (58) | 7 (41)  15 (63) | 10 (59)  9 (37) | 0.151 |
| Involved in SCLC research last 2 years  Yes  No | 8 (20)  33 (80) | 4 (50)  18 (55) | 4 (50)  15 (45) | 0.562 |
| NSCLC/SCLC patients treated annually  ≤20  >20 | 2 (5)  39 (95) | 1 (50)  21 (54) | 1 (50)  18 (46) | 0.718 |
| Number of multidisciplinary teams participating  ≤2  >2 | 21 (51)  20 (49) | 12 (57)  10 (50) | 9 (43)  10 (50) | 0.442 |
| Percentage of daily practice associated with lung cancer  ≤50%  >50% | 28 (68)  13 (32) | 15 (54)  7 (54) | 13 (46)  6 (46) | 0.626 |
| Number of patients treated with SCLC annually  1-10  >10 | 21 (51)  20 (49) | 11 (53)  11 (55) | 10 (47)  9 (45) | 0.558 |
| Year of taking note of Takahashi trial  ≤2017  2018-present | 28 (68)  12 (29) | 17 (61)  4 (33) | 11 (39)  8 (67) | 0.107 |
| Sufficient capacity of brain MRI  Yes  No | 27 (66)  14 (34) | 13 (48)  9 (64) | 14 (52)  5 (36) | 0.258 |
| Pre-chemotherapy brain MRI  Yes  No | 37 (90)  4 (10) | 21 (57)  1 (25) | 16 (43)  3 (75) | 0.249 |
| Post-chemotherapy brain MRI  Yes  No | 4 (10)  37 (90) | 1 (25)  21 (57) | 3 (75)  16 (43) | 0.249 |

| **Supplemental Table 2.3** Associations of demographic factors and MRI capacity for influence of  Takahashi in ES-SCLC | | | | |
| --- | --- | --- | --- | --- |
|  | **No. (%)** |  | |  |
|  |  | **Influence of Takahashi in daily practice regarding ES-SCLC (n=40)** | |  |
| **Factor** | **Respondents** | **Yes** | **No** | ***p* value** |
| Type of hospital  Academic  Non academic | 22 (55)  18 (45) | 15 (68)  10 (56) | 7 (32)  8 (44) | 0.517 |
| No. of ROs at location  ≤20  >20 | 21 (52)  19 (48) | 11 (52)  14 (74) | 10 (48)  5 (26) | 0.204 |
| No. of lung ROs at location  ≤4  >4 | 11 (28)  29 (72) | 4 (36)  21 (72) | 7 (64)  8 (28) | 0.065 |
| Years of experience  ≤10 years  >10 years | 17 (43)  23 (57) | 13 (77)  12 (52) | 4 (23)  11 (48) | 0.187 |
| Involved in SCLC research last 2 years  Yes  No | 8 (20)  32 (80) | 6 (75)  19 (59) | 2 (25)  13 (41) | 0.686 |
| NSCLC/SCLC patients treated annually  ≤20  >20 | 2 (5)  38 (95) | 2 (100)  23 (61) | 0 (0)  15 (39) | 0.519 |
| Number of multidisciplinary boards participating  ≤2  >2 | 20 (50)  20 (50) | 12 (60)  13 (65) | 8 (40)  7 (35) | 1.000 |
| Percentage of daily practice associated with lung cancer  ≤50%  >50% | 27 (68)  13 (32) | 16 (59)  9 (69) | 11 (41)  4 (31) | 0.730 |
| Number of patients treated with SCLC annually  1-10  >10 | 20 (50)  20 (50) | 14 (70)  11 (55) | 6 (30)  9 (45) | 0.514 |
| Year of taking note of Takahashi trial  ≤2017  2018-present | 28 (70)  12 (30) | 19 (68)  6 (50) | 9 (32)  6 (50) | 0.311 |
| Sufficient capacity of brain MRI  Yes  No | 24 (60)  16 (40) | 18 (75)  7 (44) | 6 (25)  9 (56) | 0.094 |
| Pre-chemotherapy MRI  Yes  No | 15 (37)  25 (63) | 11 (73)  14 (56) | 4 (27)  11 (44) | 0.329 |
| Post-chemotherapy MRI  Yes  No | 3 (8)  37 (92) | 3 (100)  22 (60) | 0 (0)  15 (40) | 0.279 |

| **Supplemental Table 2.4** Associations of demographic factors and MRI capacity for influence of  Takahashi in LS-SCLC | | | | |
| --- | --- | --- | --- | --- |
|  | **No. (%)** |  | |  |
|  |  | **Influence of Takahashi in daily practice regarding LS-SCLC (n=40)** | |  |
| **Factor** | **Respondents** | **Yes** | **No** | ***p* value** |
| Type of hospital  Academic  Non academic | 22 (55)  18 (45) | 6 (27)  4 (22) | 16 (73)  14 (78) | 1.000 |
| No. of ROs at location  ≤20  >20 | 21 (52)  19 (48) | 4 (19)  6 (32) | 17 (81)  13 (68) | 0.473 |
| No. of lung ROs at location  ≤4  >4 | 11 (28)  29 (72) | 1 (9)  9 (31) | 10 (91)  20 (69) | 0.233 |
| Years of experience  ≤10 years  >10 years | 17 (43)  23 (57) | 6 (35)  4 (17) | 11 (57)  19 (83) | 0.274 |
| Involved in SCLC research last 2 years  Yes  No | 8 (20)  32 (80) | 1 (13)  9 (28) | 7 (87)  23 (72) | 0.653 |
| NSCLC/SCLC patients treated annually  ≤20  >20 | 2 (5)  38 (95) | 1 (50)  9 (24) | 1 (50)  29 (76) | 0.442 |
| Number of multidisciplinary boards participating  ≤2  >2 | 20 (50)  20 (50) | 3 (15)  7 (35) | 17 (85)  13 (65) | 0.273 |
| Percentage of daily practice associated with lung cancer  ≤50%  >50% | 27 (68)  13 (32) | 9 (33)  1 (8) | 18 (67)  12 (92) | 0.124 |
| Number of patients treated with SCLC annually  1-10  >10 | 20 (50)  20 (50) | 8 (40)  2 (10) | 12 (60)  18 (90) | 0.065 |
| Year of taking note of Takahashi trial  ≤2017  2018-present | 28 (70)  12 (30) | 6 (21)  4 (33) | 22 (79)  8 (67) | 0.451 |
| Sufficient capacity of brain MRI  Yes  No | 26 (65)  14 (35) | 10 (38)  0 (0) | 16 (62)  14 (100) | **0.007*** |
| Pre-chemotherapy brain MRI  Yes  No | 36 (90)  4 (10) | 7 (19)  3 (75) | 29 (81)  1 (25) | **0.042*** |
| Post-chemotherapy brain MRI  Yes  No  ***statistically significant** | 4 (10)  36 (90) | 3 (75)  7 (19) | 1 (25)  29 (81) | **0.042*** |

| **Supplemental file 2.5** Sensitivity test of significant associations | | | |
| --- | --- | --- | --- |
|  | **Influence of Takahashi in daily practice regarding LS-SCLC (n=40)** | |  |
|  | **Yes weighted No. (%)** | **No weighted No. (%)** | ***p value*** |
| Sufficient capacity of brain MRI among LS-SCLC  Yes  No | 3.667 (34)  0 (0) | 7.16 (66)  7.16 (100) | **0.022*** |
| Pre-chemotherapy baseline brain MRI among LS-SCLC  Yes  No | 2.5 (15)  1.167 (87) | 14.167 (85)  0.167 (13) | 0.124 |
| Post-chemotherapy baseline brain MRI among LS-SCLC  Yes  No | 0.83 (72)  2.83 (17) | 0.33 (28)  14.0 (83) | 0.219 |

***statistically significant**
